# Gray-level discretization impacts reproducible MRI radiomics texture features

**DOI:** 10.1371/journal.pone.0213459

**Published:** 2019-03-07

**Authors:** Loïc Duron, Daniel Balvay, Saskia Vande Perre, Afef Bouchouicha, Julien Savatovsky, Jean-Claude Sadik, Isabelle Thomassin-Naggara, Laure Fournier, Augustin Lecler

**Affiliations:** 1 Department of Radiology, Fondation Ophtalmologique Adolphe de Rothschild, Paris, France; 2 Université Paris Descartes Sorbonne Paris Cité, INSERM UMR-S970, Cardiovascular Research Center—PARCC, Paris, France; 3 Sorbonne Universités, UPMC Univ Paris 06, Institut Universitaire de Cancérologie, Assistance Publique-Hôpitaux de Paris (AP-HP), Hôpital Tenon, Service d'Imagerie, 4 rue de la Chine, Paris, France; 4 Université Paris Descartes Sorbonne Paris Cité, Assistance Publique-Hôpitaux de Paris, Hôpital Européen Georges Pompidou, Radiology Department, Paris, France; University of Pennsylvania Perelman School of Medicine, UNITED STATES

## Abstract

**Objectives:**

To assess the influence of gray-level discretization on inter- and intra-observer reproducibility of texture radiomics features on clinical MR images.

**Materials and methods:**

We studied two independent MRI datasets of 74 lacrymal gland tumors and 30 breast lesions from two different centers. Two pairs of readers performed three two-dimensional delineations for each dataset. Texture features were extracted using two radiomics softwares (Pyradiomics and an in-house software). Reproducible features were selected using a combination of intra-class correlation coefficient (ICC) and concordance and coherence coefficient (CCC) with 0.8 and 0.9 as thresholds, respectively. We tested six absolute and eight relative gray-level discretization methods and analyzed the distribution and highest number of reproducible features obtained for each discretization. We also analyzed the number of reproducible features extracted from computer simulated delineations representative of inter-observer variability.

**Results:**

The gray-level discretization method had a direct impact on texture feature reproducibility, independent of observers, software or method of delineation (simulated vs. human). The absolute discretization consistently provided statistically significantly more reproducible features than the relative discretization. Varying the bin number of relative discretization led to statistically significantly more variable results than varying the bin size of absolute discretization.

**Conclusions:**

When considering inter-observer reproducible results of MRI texture radiomics features, an absolute discretization should be favored to allow the extraction of the highest number of potential candidates for new imaging biomarkers. Whichever the chosen method, it should be systematically documented to allow replicability of results.

## Introduction

Medical imaging is progressively shifting from conventional visual image analysis to quantitative personalized medicine thanks to the recent development of data-driven analysis methods like radiomics [1]. Radiomics is a high-throughput mining of quantitative features from medical imaging that enables data to be extracted and applied within clinical-decision support systems to improve diagnostic, prognostic, and predictive accuracy [2]. The potential of radiomics-based phenotyping in precision medicine is encouraging [2–4] but the diversity of the implementation methods of the radiomics pipeline and the absence of widespread standards result in a high variability of the possible approaches that may lead to non-replicable results. More specifically, one of the steps in the radiomics process is feature reduction, to decrease data dimensionality and allow correlation of imaging features to predict outcome. Feature reduction can be performed in a number of ways, but a frequently-used method is based on the selection of the most reproducible features, based on the hypothesis that reproducibility is a mandatory quality for an imaging biomarker derived from the radiomics process. This feature-reduction step will therefore impact the results of a radiomics study, and may lead to potentially discard a highly informative feature because it is not reproducible enough to be used in clinical routine. However, there are no current recommendations or standardization of this step.

Radiomics texture features are calculated from gray-level co-occurrence matrices and from other derived matrices that are computed on images after a gray-level discretization. The gray-level discretization consists in clustering pixels according to intensity values to facilitate the calculation of texture features [5]. Two approaches to discretization are commonly used. The first, called relative discretization, involves clustering of the pixels in the image to a fixed number of bins (FBN method); the other, called absolute discretization, uses a fixed bin size (FBS method). The gray-level discretization has been shown to substantially impact feature values extracted from PET [6] and CT [7,8] images. However, few studies have addressed the effect of gray-level discretization of clinical MR images [9]. Particularly, its effect on texture feature reproducibility is unknown.

In this context, the purpose of this work was to investigate how pre-processing of images, and specifically gray-level discretization methods, could impact the number and type of reproducible texture features extracted from clinical MR images.

## Materials and methods

### Study design

This study was performed on two datasets: the DATASET 1 was prospectively acquired in a tertiary referral center specializing in ophthalmic diseases with the approval of the Institutional Research Ethics Board, of the Ile-De-France Ethics Committee (France) and adhered to the tenants of the Declaration of Helsinki (IRB 2015-A00364-45, NCT02401906); signed informed consent was obtained from all subjects. The DATASET 2 was retrospectively built in a tertiary referral center specializing in breast lesions with the approval of the local Ethics Committee of Tenon Hospital (Paris, France) and the Institutional Review Board (IRB “blinded”), which waived the necessity for informed consent. Information was given to all patients.

### Datasets description and MRI acquisition protocol

Two datasets of medical MR images were acquired in two distinct centers:

- The DATASET 1 consisted in multi-parametric MR images of 74 lacrymal gland lesions prospectively collected from December 2015 to April 2017 in a tertiary referral center specializing in ophthalmic diseases, using a 3 Tesla Philips INGENIA device with a 32-channel head coil (Philips Medical Systems, Best, Netherlands). The MRI protocol, which included 6 MR sequences, is detailed in S1 Table.
- The DATASET 2 consisted in MR images of 30 breast lesions prospectively collected from November 2017 to April 2018 on a 1.5 Tesla General Electric MR scanner using a phased array dedicated breast coil covering both breasts. Patients were imaged in the prone position. The sequence of interest was an ultrafast dual-echo 3D spoiled gradient recalled acquisition sequence called DISCO (Differential Subsampling With Cartesian Ordering), with pseudorandom variable density k-space segmentation, with an elliptical model where the central k-space regions were acquired more frequently than the peripheral region and a view-sharing reconstruction. The MRI acquisition parameters are detailed in S1 Table. This sequence was performed before and eleven times during one minute and twenty second after bolus injection of Gadolinium chelate (Dotarem; Guerbet, France) (0,1mmol.kg-1 body weight) given by a power injector (Medrad, Maastrich, Netherlands) at a rate of 2ml.s-1 followed by 20ml of saline flush. The fifth post-injection series was arbitrarily chosen for radiomics feature extraction.

### Manual segmentation

#### DATASET 1

Two readers reviewed the whole imaging DATASET 1, a senior neuro-radiologist specialized in orbital imaging with 8 years of experience (*blinded*) and a junior radiologist with 6 months of experience (*blinded*), blinded to patient ID, medical history, lab results and pathological results. Each reader independently performed a 2D manual delineation of the two lacrimal glands of each patient on each MR sequence. The slice of delineation was independently chosen by each reader, considered as the slice where the lesion had the largest diameter. The delineation included both the tumor and the normal glands. A total of 444 ROIs were drawn by each reader (2 lacrimal glands per patient on 6 MR sequences for 37 patients). One reader (*blinded*) performed a second segmentation session to assess the intra-observer reproducibility. The segmentations were performed using an in-house software developed on Matlab (Matlab R2013b, The Mathworks, Natick, MA, USA) and dedicated to the radiomics analysis [10].

#### DATASET 2

Two junior radiologists reviewed the whole imaging DATASET 2 (*blinded*, *blinded*, both 6 months of experience) blinded to patient ID, medical history, lab results and pathological results. Each reader independently performed a 2D manual delineation of the breast lesions on the DISCO sequence. The slice of delineation was independently chosen by each reader, considered as the slice where the lesion had the largest diameter. One reader (*blinded*) performed a second segmentation session to assess the intra-observer reproducibility. The segmentations were performed using the ITK-SNAP software (Yushkevich et al., version 3.6.0) [11].

### Computer-simulated segmentation

**Fig 1:**
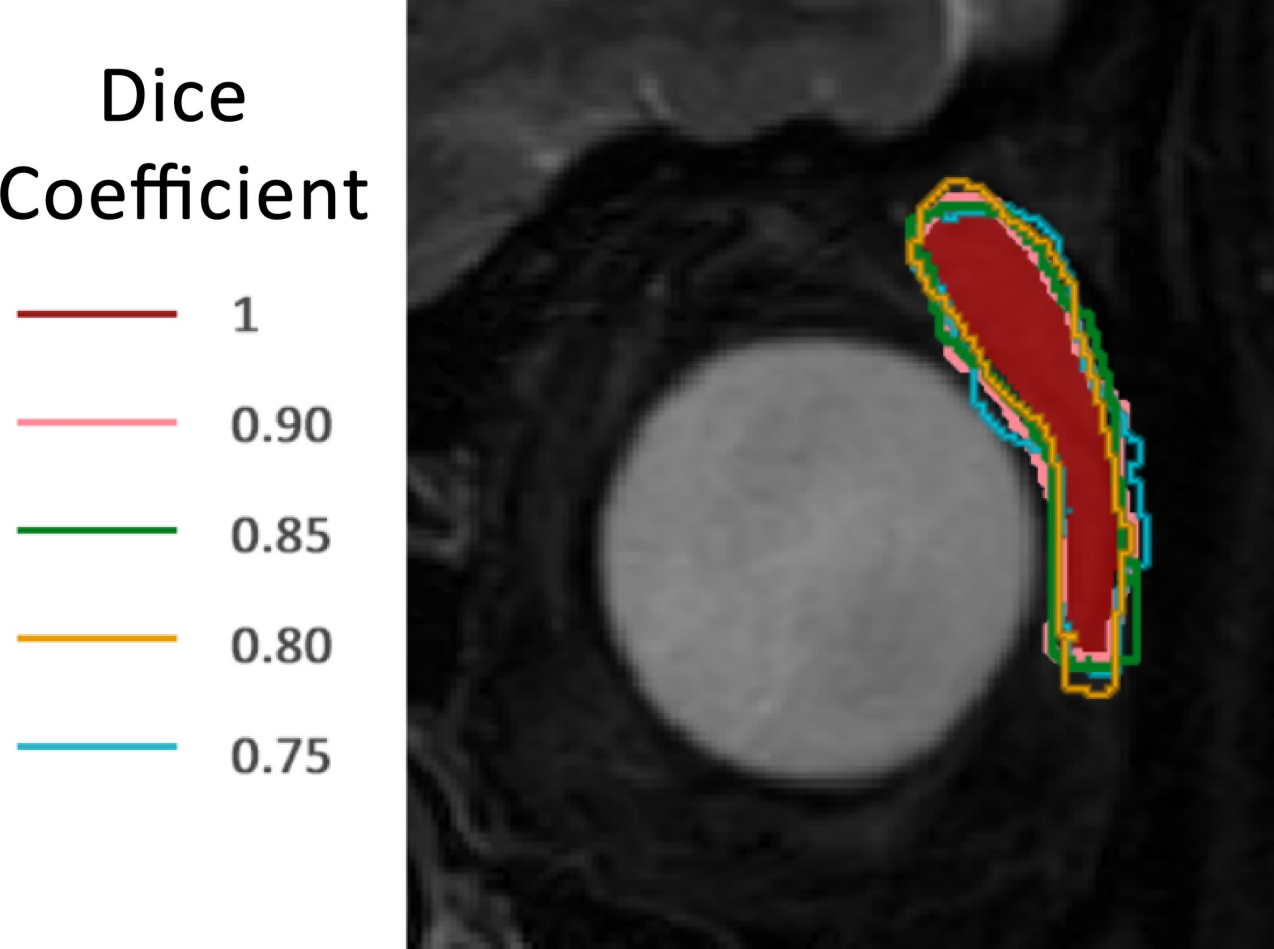
Example of computer simulations of delineations. The reference manual delineation is in red. Computer simulations with corresponding Dice coefficient are superimposed.

To overcome the potential bias of the small number of readers that may not represent the true inter- and intra-observer segmentation variability, we developed a computer simulation of a high number of delineations (5000) based on morphological binary openings, closings and elastic transforms applied to a manual reference delineation for each ROI of DATASET 1. The Dice coefficient, which is a measurement of similarity between two segmentations, was calculated between each simulated ROI and the human reference one. Then, one simulated segmentation per Dice coefficient interval of 0.05 from 1.0 (no transformation) to 0.65 (maximum tested transformation) was randomly selected. This interval from 1.0 to 0.65 was chosen to encompass the whole variability different manual segmentations may provide. This led to 7 randomly selected simulated ROIs for each associated human reference ROI with Dice coefficients ranging from 0.65 to 0.95. The segmentations simulations were visually checked to make sure it was consistent with a potential manual delineation variability. An example of computer simulations is given in Fig 1.

### Gray-level discretization methods

Two methods of discretization were used:

- An absolute discretization with fixed bin size (FBS method), where a new bin is assigned to pixel intensities each BS gray level, starting from 0, according to the following Eq 1:

$$I_{BS}(x)=\left\lceil \frac{I(x)}{BS}\right\rceil -min\left(\lceil \frac{I(x)}{BS}\rceil \right)+1$$ (1)

Where I(x) is the intensity of voxel x, BW the bin size and I_BS_(x) the discretized gray-level of voxel x. The term [min(I(x)/BS) + 1] ensures that the bin count starts at 1. We tested six different bin sizes: 1, 5, 10, 20, 25 and 50.

- A relative discretization with fixed bin number (FBN method), starting from the minimum intensity value of the segmented area and defined as follows:

$$I_{BN}(x)=\left\{ \begin{array}{l} \;1\;if\;I(x)=\min\left(I(x)\right)\\ \lceil BN*\frac{I(x)-min(I(x))}{\max\left(I(x)\right)-min(I(x))}\rceil \;otherwise \end{array}\right.$$ (2)

Where I(x) is the intensity of voxel x, BN the bin number and I_BN_(x) the discretized gray-level of the voxel x. We tested eight different fixed bin numbers: 8, 16, 32, 64, 128, 256, 512 and 1024.

### Feature extraction

We performed the feature extraction for each discretization using two radiomics-dedicated softwares:

- Pyradiomics open-source software (*Griethuysen et al*., version 1.3.0) [12] was used on DATASET 1 and DATASET 2 to extract texture features. No pixel resampling nor filter was applied to the images. Each ROI provided 69 texture features, derived from the gray-level co-occurrence matrix (GLCM, 23 features) obtained using 4 angles, gray-level run-length matrix (GLRLM, 16 features), gray-level size-zone matrix (GLSZM, 16 features) and gray-level dependence matrix (GLDM, 14 features). A total of 30 636 texture feature values for each discretization was calculated for DATASET 1 and 2 070 for DATASET 2.
- An in-house software developed on Matlab (Matlab R2013b, The Mathworks, Natick, MA, USA) and dedicated to the radiomics analysis [10] was also used to performed feature extraction on DATASET 1. Each ROI provided 57 texture features derived from the gray-level co-occurrence matrix (GLCM, 26 features) obtained using 4 angles, gray-level run-length matrix (GLRLM, 13 features), gray-level size-zone matrix (GLSZM, 13 features) and neighborhood gray-tone difference matrix (NGTDM, 5 features). A total of 25 308 texture feature values for each discretization was calculated.
- Texture features extracted are listed in S2 Table. Their mathematical definitions are available elsewhere [5,12]. Detailed extraction parameters are given in S3 Table.

### Feature reduction according to intra- and inter-observer reproducibility

In order to select features that are reproducible between observer delineations, we performed a 2-way random intraclass correlation coefficient (ICC) (absolute agreement, average type) on pair combinations of readings (1/2, 1/3, 2/3) and a Lin’s concordance correlation coefficient (CCC) on the intra-observer pair. A feature was considered as highly reproducible and therefore selected if all three ICC values were above 0.8 and the CCC value was above 0.9. All other features were eliminated.

### Data analysis

#### Experiment 1—Reproducible features using Pyradiomics on DATASET 1 according to the gray-level discretization

For each discretization method and each MR sequence of DATASET 1, we analyzed the highest number of reproducible texture features obtained from the Pyradiomics software with FBS versus FBN discretization methods, using an exact Fisher test. We also assessed if there was a difference in reproducible feature distributions among chosen bin sizes for FBS or among chosen bin numbers for FBN, using Chi-square tests.

#### Experiment 2—Reproducible features using an in-house Matlab-based software on DATASET 1 according to the gray-level discretization

For each discretization method and each MR sequence, we repeated the same analysis on the data extracted from the DATASET 1 using our in-house feature extraction software.

#### Experiment 3—Reproducible features using Pyradiomics on DATASET 2 according to the gray-level discretization

For each discretization method, we repeated the same analysis using the Pyradiomics software on DATASET 2.

#### Experiment 4—Reproducible features using Pyradiomics and computer simulated delineations on DATASET 1 according to the gray-level discretization

Texture features were extracted from each Dice coefficient associated ROI as described in the *Feature Extraction* paragraph, using the Pyradiomics software.

We focused on the simulated ROIs associated with a Dice coefficient of 0.85 because the mean Dice coefficient of manual delineations of pooled sequences of DATASET 1 (Experiment 1) was 0.84. We performed feature reduction according to the delineation variability by selecting features for which the ICC between the reference human ROI (Dice 1) and the simulated ROI (Dice 0.85) was above 0.8. We then performed the same analysis as described in previous sections.

We also analyzed the variation of the highest number of reproducible features according to the Dice coefficient for FBS methods versus FBN methods.

**Fig 2:**
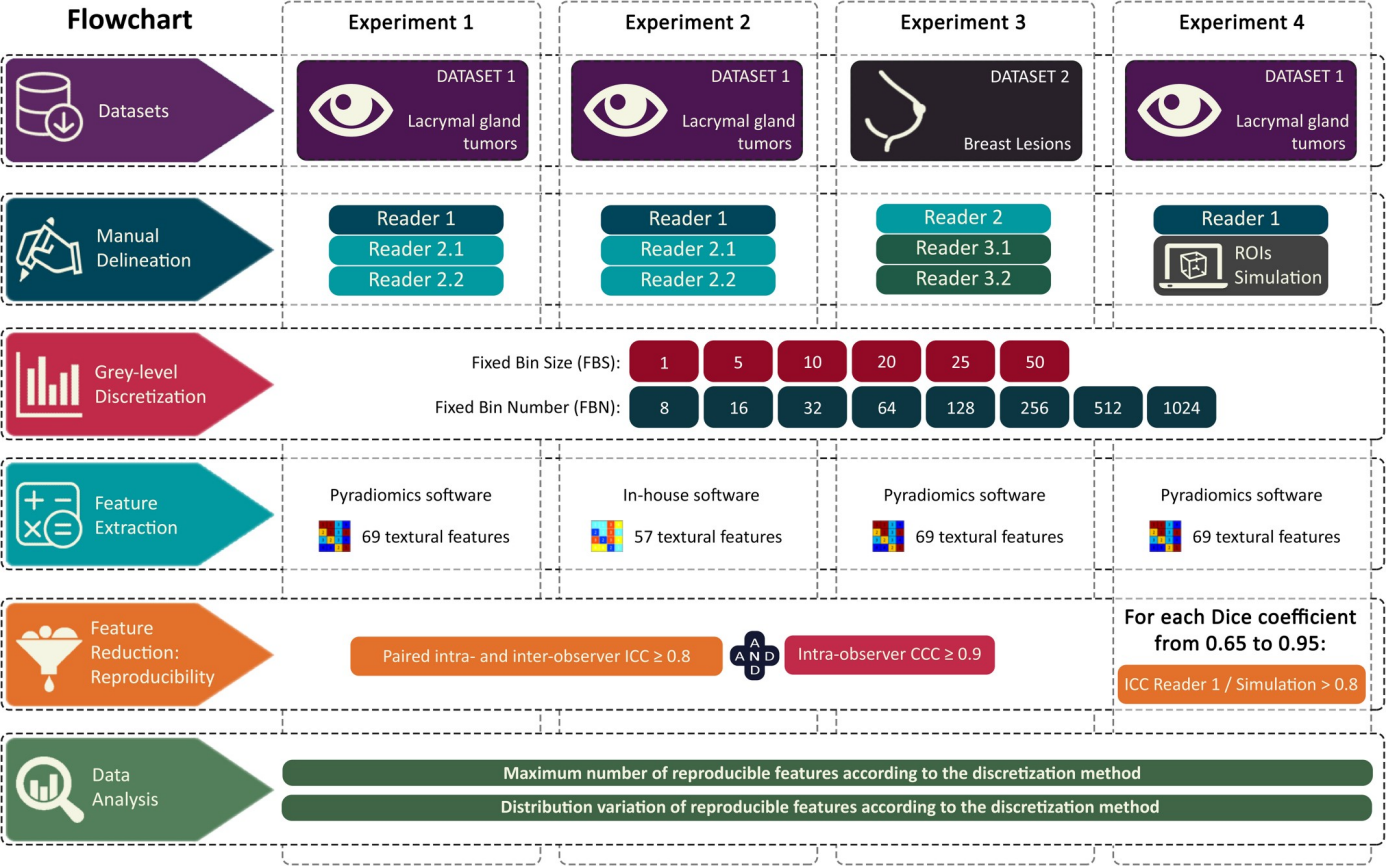
Flowchart of the study. Reader 2.1 = Reader 2, delineation session 1; Reader 2.2 = Reader 2, delineation session 2; Reader 3.1 = Reader 3, delineation session 1; Reader 3.2 = Reader 3, delineation session 2; ROIs = Regions Of Interest; ICC = Intra-class correlation coefficient; CCC = Concordance correlation coefficient.

The flowchart of the study is detailed in Fig 2.

### Statistical analyses

All feature extractions and discretization methods were implemented using Python language for Pyradiomics software and Matlab language for our in-house feature extraction software. All statistical analyses of extracted data were performed using R-3.3.3 (R Foundation, Vienna, Austria) [13]. P -value ≤ 0.05 was considered statistically significant.

## Results

The results of the experiments 1, 2, 3 and 4 are detailed in the Tables 1, 2, 3 and 4, respectively.

**Table 1:** Reproducible features using the Pyradiomics software and manual delineations on DATASET 1 according to the gray-level discretization.

Sequence	Difference in reproducible features distribution (p-value)		Highest number of reproducible features		
	Among FBS	Among FBN	Number		P-Value
			FBS	FBN	
**wDIXON-T2-WI**	0.56	< 0.0001*	**54**	32	< 0.001*
**ipDIXON-T2-WI**	0.17	< 0.001*	**47**	32	< 0.05*
**pc wDIXON-T1-WI**	0.96	< 0.05*	**37**	34	0.73
**pc ipDIXON-T1-WI**	1	< 0.01*	**35**	33	0.86
**T1-WI**	< 0.01*	< 0.0001*	**21**	21	1
**ADC map**	1	0.41	**31**	9	< 0.001*

FBS = Fixed Bin Size (absolute discretization), FBN = Fixed Bin Number (relative discretization). ***Sequence abbreviations*:** Water DIXON-T2-WI (wDIXON-T2-WI); In-Phase DIXON-T2-WI (ipDIXON-T2-WI); Post-Contrast Water DIXON-T1-WI (pc wDIXON-T1-WI); Post-Contrast In-Phase DIXON-T1-WI (pc ipDIXON-T1-WI); Apparent Diffusion Coefficient map (ADC map).

* = statistically significantly different among discretizations.

**Table 2:** Reproducible features using the in-house Matlab-based software and manual delineations on DATASET 1 according to the gray-level discretization method.

Sequence	Difference in reproducible features distribution (p-value)		Highest number of reproducible features		
	Among FBS	Among FBN	Number		P-Value
			FBS	FBN	
**wDIXON-T2-WI**	0.15	< 0.001*	**22**	15	0.23
**ipDIXON-T2-WI**	0.52	0.17	**15**	7	0.09
**pc wDIXON-T1-WI**	0.98	< 0.001*	**17**	14	0.67
**pc ipDIXON-T1-WI**	0.98	0.29	**16**	8	0.11
**T1-WI**	0.79	< 0.0001*	4	**13**	NA
**ADC map**	0.95	0.76	**6**	5	1

FBS = Fixed Bin Size (absolute discretization), FBN = Fixed Bin Number (relative discretization). ***Sequence abbreviations*:** Water DIXON-T2-WI (wDIXON-T2-WI); In-Phase DIXON-T2-WI (ipDIXON-T2-WI); Post-Contrast Water DIXON-T1-WI (pc wDIXON-T1-WI); Post-Contrast In-Phase DIXON-T1-WI (pc ipDIXON-T1-WI); Apparent Diffusion Coefficient map (ADC map).

* = statistically significantly different among discretizations.

**Table 3:** Reproducible features using the Pyradiomics software and manual delineations on DATASET 2 according to the gray-level discretization.

Sequence	Difference in reproducible features distribution (p-value)		Highest number of reproducible features		
	Among FBS	Among FBN	Number		P-Value
			FBS	FBN	
**DISCO sequence**	0.54	0.25	**32**	17	< 0.05*

FBS = Fixed Bin Size (absolute discretization), FBN = Fixed Bin Number (relative discretization). ***Sequence abbreviations*:** DISCO = Differential Subsampling With Cartesian Ordering.

* = statistically significantly different among discretizations.

**Table 4:** Reproducible features using the Pyradiomics software and simulated delineations with a Dice coefficient of 0.85 on DATASET 1 according to the gray-level discretization.

Sequence	Difference in reproducible features distribution (p-value)		Highest number of reproducible features		
	Among FBS	Among FBN	Number		P-Value
			FBS	FBN	
**wDIXON-T2-WI**	0.14	< 0.0001*	**57**	37	< 0.001*
**ipDIXON-T2-WI**	0.69	0.75	**62**	46	< 0.01*
**pc wDIXON-T1-WI**	0.28	< 0.001*	**66**	57	< 0.05*
**pc ipDIXON-T1-WI**	0.41	< 0.01*	**64**	59	0.27
**T1-WI**	0.14	< 0.05*	**54**	44	0.09
**ADC map**	1	0.25	**43**	26	< 0.05*

FBS = Fixed Bin Size (absolute discretization), FBN = Fixed Bin Number (relative discretization). ***Sequence abbreviations*:** Water DIXON-T2-WI (wDIXON-T2-WI); In-Phase DIXON-T2-WI (ipDIXON-T2-WI); Post-Contrast Water DIXON-T1-WI (pc wDIXON-T1-WI); Post-Contrast In-Phase DIXON-T1-WI (pc ipDIXON-T1-WI); Apparent Diffusion Coefficient map (ADC map).

* = statistically significantly different among discretizations.

**Fig 3:**
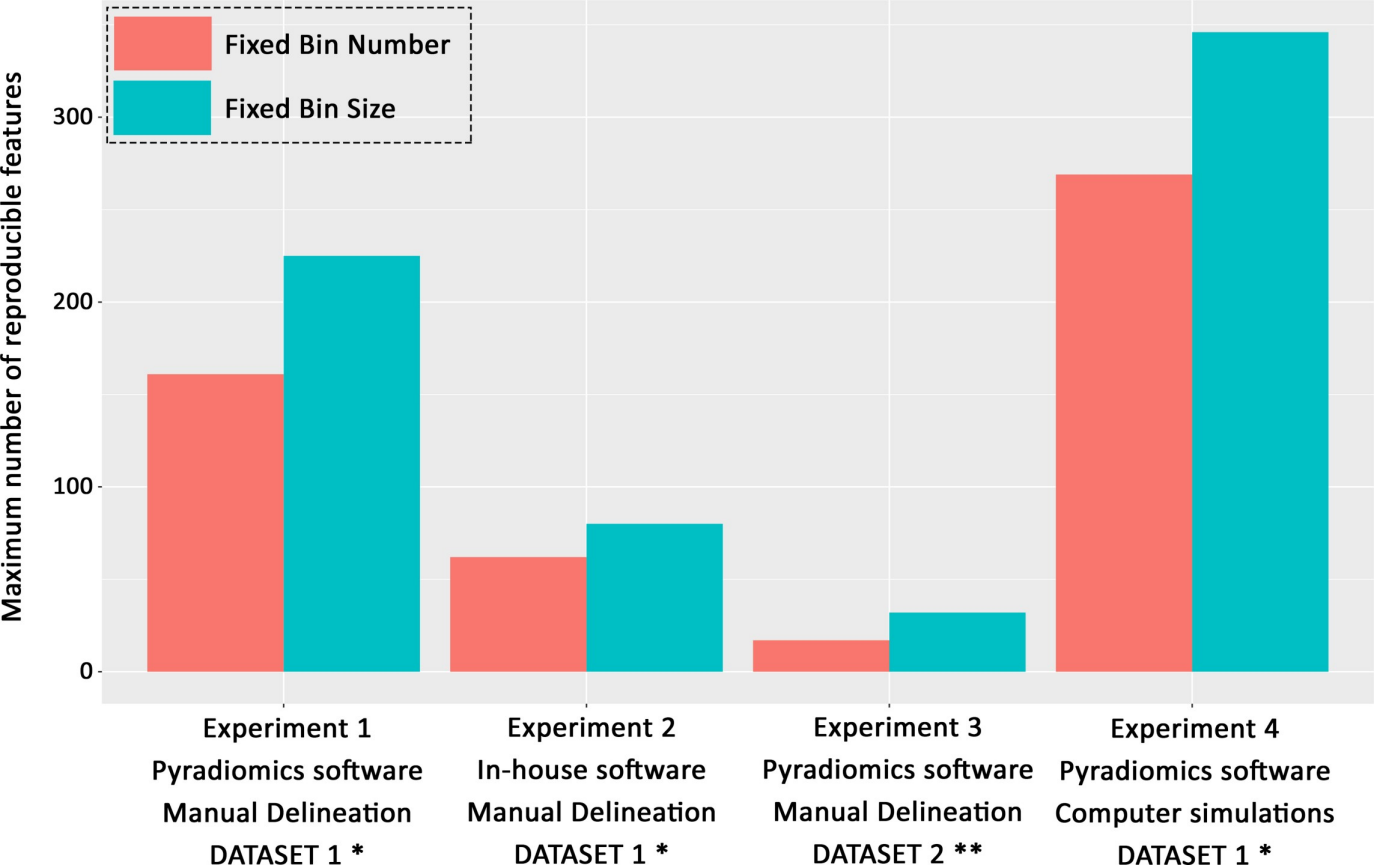
Highest number of reproducible texture features obtained for each experiment according to the discretization method. FBS = Fixed Bin Size (absolute discretization); FBN = Fixed Bin Number (relative discretization). * 6 MR sequences; ** 1 MR sequence.

In each of the above experiments, the gray-level discretization method impacted both the number and the distribution of reproducible features observed. Overall, the highest number of reproducible features was consistently obtained using FBS methods. The difference between FBS and FBN methods was significant in most cases. An illustration of the highest number of reproducible texture features for the FBS and FBN methods in each experiment is given in Fig 3. The distributions of reproducible features were more often statistically different when varying the bin number among tested FBN methods than when varying bin sizes among tested FBS methods. The highest number of reproducible features was obtained using large bin numbers (512 or 1024) or small bin widths (1, 5, 10, or 20) in all cases.

**Fig 4:**
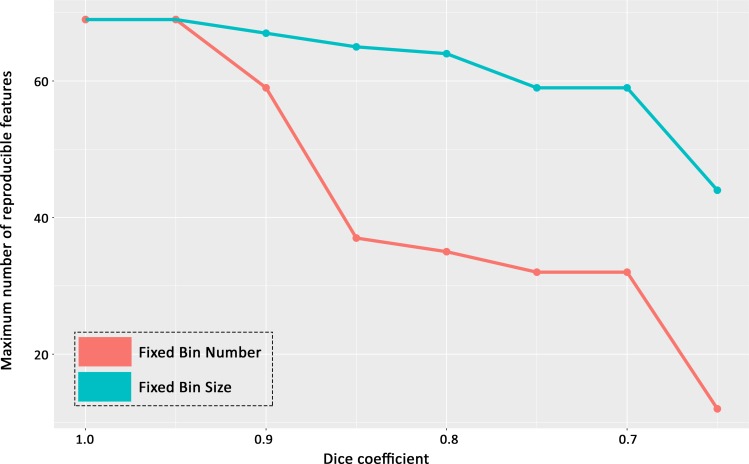
Highest number of reproducible texture features using simulated ROIs on DATASET 1 with the Pyradiomics software according to the Dice coefficient ranging from 1.0 to 0.65, for FBS versus FBN methods. FBS = Fixed Bin Size (absolute discretization); FBN = Fixed Bin Number (relative discretization).

The highest number of reproducible features extracted from computer simulated delineations decreased according to the Dice coefficient from 1 to 0.65. The number of reproducible features obtained from the FBS method was much less impacted by variability of ROI delineation than the number obtained by the FBN method, and the difference between FBS and FBN methods increased with the decrease of the Dice coefficient. The results for the water DIXON-T2-WI are illustrated in Fig 4. The difference in the highest number of reproducible features according to the discretization method (FBS versus FBN methods) was statistically significant for all Dice coefficients lesser or equal to 0.9 (p < 0.05).

Whatever the discretization method, the DATASET and the MR sequence, the three most reproducible features were: GLRLM Gray-Level Non-Uniformity, GLDM Gray-Level Non-Uniformity, GLDM Dependence Non-Uniformity. The three least reproducible features were: GLDM Large Dependence Low Gray-Level Emphasis, GLCM Cluster Shade and GLRLM Long Run Low Gray-Level Emphasis.

## Discussion

We assessed the influence of gray-level discretization methods on radiomics texture feature inter- and intra-observer reproducibility in two independent clinical MR datasets. Texture feature reproducibility was shown to substantially depend on the gray-level discretization method. The absolute discretization (FBS) method was found to provide a higher number of reproducible features than the relative discretization (FBN) method. The relative discretization was also found to give more different results when varying the bin numbers than the absolute discretization when varying the bin sizes.

Although the gray-level discretization has been assessed in PET and CT studies [6–8], few studies have focused on MR images. Molina & al. [9] assessed the variability of texture feature values on post-contrast T1-WI in three patients with glioblastomas and one patient with a non-small cell lung cancer brain metastasis, using a FBN method with 8, 16, 32 and 64 gray levels. They found that modifying the discretization parameters led to significantly different results. More recently, Goya-Outi et al. [14] investigated the impact of the absolute and relative discretization methods on patient ranking compared to the visual assessment of the heterogeneity of diffuse intrinsic pontine gliomas on 30 patients and 4 MR sequences (T1-WI, post-contrast T1-WI, T2-WI and Fluid Attenuation Inversion Recovery (FLAIR)). They concluded that using an absolute (FBS) discretization method provided more consistent results compared to the visual assessment of the heterogeneity. The Image Biomarker Standardization Initiative (IBSI) [5] has recently proposed guidelines to help select the best gray-level discretization for each purpose. The recommendations were to use relative discretization when dealing with MR raw data, though no reference is given to support this recommendation. To the best of our knowledge, no study has investigated the influence of absolute and relative discretization on multiple MR sequences, multiple datasets and multiple softwares.

We assessed the influence of gray-level discretization on the inter and intra-observer reproducibility of texture features because most of the radiomics studies currently integrate the inter and intra-observer reproducibility as a data reduction step to select robust features and exclude all others [7,15–17]. This step, and how it is performed, will therefore greatly impact the features that may emerge from the radiomics process as potential imaging biomarkers.

Our results may be explained by the calculation process used to perform relative and absolute discretization methods. When using relative discretization (FBN), the intensity range of the segmented image will impact the bin size, which in turn determines the intensity histogram, the co-occurrence and other derived matrices used to compute texture features. Most of the inter and intra-observer segmentation variability is related to slightly different contours providing intensity outliers, thereby changing the intensity range of the region of interest. Conversely, the absolute discretization (FBS) method is independent from the intensity range of the segmented image and so may be less sensitive to inter- and intra-observer delineation variability.

We used two independent and very different datasets for external validation, two pairs of readers independently delineating the datasets, computer simulations of manual delineation variability, two delineation softwares to get rid of the delineation software confounding factor, and two radiomics softwares for which the calculation method of gray-level co-occurrence matrices and other derived matrices was slightly different. The purpose was to determine whether our results applied to different data, quantification methods, or organs. The fixed bin size method of gray-level discretization consistently provided less variable results. There are currently no specific guidelines to address the choice of the best bin width or bin number. Available studies use bin numbers varying from 8 to 1000, as suggested by the IBSI [5,14], or bin widths from 1 to 75 [14,18]. This allows for differing ranges of signal intensity in ROIs, while still keeping the texture features informative and comparable between lesions. The ideal choice probably depends on the target lesions and the wish to enhance coarse or fine textures. We tested a large panel of bin widths and bin numbers to consider the largest possible choices and not limit our conclusions to a selected range of bin widths and/or numbers.

This study has some limitations. We did not normalize signal intensity on the MR images before extracting texture features. The IBSI recommends to use the normalization on raw MR data because it is based on arbitrary units. The normalization may impact texture feature values, especially when dealing with multiple MR machines and when comparing feature values among patients [14,19,20]. However, we focused on the reproducibility of texture features between observers and not between patients. Therefore, for a given patient, the signal intensity values were constant in the image and the range in the ROI was impacted only by the difference in delineations. When performed after intensity normalization, the experiment 1 yielded comparable results, as detailed in S4 Table. Many other sources of variability may impact the results of the radiomics pipeline and should be investigated, including acquisition parameters, denoising, spatial interpolation methods applied on images or regions of interest, texture features calculation methods. For instance, we observed significant differences in terms of number of reproducible features between radiomics softwares. As delineations and pre-processing steps were the same, two differences may explain this. First, we extracted 57 texture features per delineation with our in-house software versus 69 with Pyradiomics. Second, the calculation method of co-occurrence matrices and other derived matrices is different between the two softwares, as explained in S5 Table, both described in the IBSI guidelines. There are currently no recommendation to favor one method over another. It may be noted that the delineation of Dataset 2 was performed only by junior radiologists with 6 months of experience in breast MRI, which may impact the clinical significance of each specific ROI. However, the purpose of the study was not to have clinically exact ROIs but rather to study the impact of differences in delineations among observations. Finally, the method presented here to select inter and intra-observer reproducible features does not take into account whether the selected features were informative (i.e. predictive of an outcome). It is only one step meant to exclude non-reproducible features and keep the maximum number of robust potential candidates for new imaging biomarkers. However, the final purpose of the radiomics process is to identify potential imaging biomarkers based on their capacity to predict an outcome. We believe it is desirable to have the largest number of reproducible features so that potentially predictive features were not erroneously excluded for technical reasons.

This study showed that the gray-level discretization of MR images influences the number and distribution of inter and intra-observer reproducible texture features. We showed that using a fixed bin size method (absolute discretization) allowed preserving a higher number of reproducible features which may be potential imaging biomarkers candidates. Considering the impact of the discretization method on results, the precise method used should be clearly documented in each radiomics study to improve the replicability of the results, as recommended in the recent evaluation criteria and reporting guidelines published to improve reliability, comparability and generalizability of radiomics-based studies [2]. The standardization of reporting radiomics studies is an essential step toward the credibility and subsequent adoption of the data-driven radiomics process.

## Supporting information

S1 Table MRI acquisition protocols of DATASETS 1 and 2. The DATASET 1 included 6 MR sequences: T1 Weighted Images (WI); Apparent Diffusion Coefficient (ADC) maps calculated voxel-wise as the linear slope of signal decrease between b0 and b1000 of Diffusion Weighted Imaging (DWI) acquisitions; In-Phase and Water DIXON-T2-WI (ipDIXON-T2-WI and wDIXON-T2-WI, respectively); Post-contrast In-Phase and Water DIXON-T1-WI (Post-contrast ipDIXON-T1-WI and wDIXON-T1-WI, respectively), obtained after administration of intravenous contrast injection of a single bolus (0.1 mmol/kg) of Gadobutrol (Gadovist; Bayer HealthCare, Berlin, Germany). The DATASET 2 included 1 MR sequence called DISCO (Differential Subsampling With Cartesian Ordering).
(DOCX)

S2 Table List of the texture features extracted using the Pyradiomics and in-house Matlab-based softwares. (DOCX)

S3 Table Feature extraction details according to the Imaging Biomarker Standardization Initiative (IBSI) guidelines. (DOCX)

S4 Table Experiment 1 results after intensity normalization. **Reproducible features using the Pyradiomics software and manual delineations on DATASET 1 according to the gray-level discretization.** The intensity normalization was performed as follows: images were centered by their mean and scaled by their standard deviation; intensity values below or above three standard deviations from the mean were excluded; intensity values were shifted to get only positive values; intensities were then scaled to the initial common range (all images of each sequence had a similar range). The normalization was not applied to the parametric ADC map.
(DOCX)

S5 Table Pyradiomics versus in-house software texture features calculation methods. Pyradiomics computes one matrix per distance and/or angle (according to the texture feature), normalizes the matrix, calculates one feature value per normalized matrix and averages the results to obtain the final texture value. Our in-house software computes a weighted sum of all non-normalized matrices obtained with the different distances/angles (diagonals are weighted by √2), then directly calculates the final feature value based on this unique matrix. These two methods are described in the IBSI guidelines. There is no current recommendation to favor one method over another.
(DOCX)
