# Supplementary material for: Gray-level discretization impacts reproducible MRI radiomics texture features
Source: PLoS One. 2019 Mar 7;14(3):e0213459. doi: 10.1371/journal.pone.0213459 (PMC6405136; doi:10.1371/journal.pone.0213459)
Supplement: S1 Table — The DATASET 1 included 6 MR sequences: T1 Weighted Images (WI); Apparent Diffusion Coefficient (ADC) maps calculated voxel-wise as the linear slope of signal decrease between b0 and b1000 of Diffusion Weighted Imaging (DWI) acquisitions; In-Phase and Water DIXON-T2-WI (ipDIXON-T2-WI and wDIXON-T2-WI, respectively); Post-contrast In-Phase and Water DIXON-T1-WI (Post-contrast ipDIXON-T1-WI and wDIXON-T1-WI, respectively), obtained after administration of intravenous contrast injection of a single bolus (0.1 mmol/kg) of Gadobutrol (Gadovist; Bayer HealthCare, Berlin, Germany). The DATASET 2 included 1 MR sequence called DISCO (Differential Subsampling With Cartesian Ordering). (DOCX) [file pone.0213459.s001.docx]

**S1 Table. MRI acquisition protocols of DATASETS 1 and 2**. The DATASET 1 included 6 MR sequences: T1 Weighted Images (WI); Apparent Diffusion Coefficient (ADC) maps calculated voxel-wise as the linear slope of signal decrease between b0 and b1000 of Diffusion Weighted Imaging (DWI) acquisitions; In-Phase and Water DIXON-T2-WI (ipDIXON-T2-WI and wDIXON-T2-WI, respectively); Post-contrast In-Phase and Water DIXON-T1-WI (Post-contrast ipDIXON-T1-WI and wDIXON-T1-WI, respectively), obtained after administration of intravenous contrast injection of a single bolus (0.1 mmol/kg) of Gadobutrol (Gadovist; Bayer HealthCare, Berlin, Germany). The DATASET 2 included 1 MR sequence called DISCO (Differential Subsampling With Cartesian Ordering).

|  | **DATASET 1 (lacrymal glands)** | | | | **DATASET 2 (breast)** |
| --- | --- | --- | --- | --- | --- |
|  | **T1-WI** | **DWI (b0-b1000)** | **ipDIXON-T2-WI and wDIXON-T2-WI** | **Post-contrast ipDIXON-T1-WI and wDIXON-T1-WI** | **DISCO** |
| **Plane** | Axial | Axial | Coronal | Coronal | Axial |
| **Number of Slices** | 20 | 15 | 30 | 15 | 160 |
| **Slice thickness (mm)** | 2.5, no gap | 3, no gap | 2, no gap | 3, no gap | 2, no gap |
| **TR (ms)** | 622 | 5750 | 3000 | 456 | 6.3 |
| **TE (ms)** | 7 | 79 | 80 | 12 | 3.3 |
| **Number of excitations** | 1 | 3 | 2 | 1 | 1 |
| **Bandwidth (Hz)** | 280 | 1048 | 260 | 376 | 125 |
| **Matrix** | 480 x 480 | 176 x 176 | 448 x 448 | 320 x 320 | 512 x 512 |
| **FOV (mm)** | 180 x 180 | 140 x 140 | 150 x 150 | 235 x 235 | 350 x 350 |
| **Acquisition duration (s)** | 80 | 184 | 246 | 235 | 7.7 |
