# Supplementary material for: Gray-level discretization impacts reproducible MRI radiomics texture features
Source: PLoS One. 2019 Mar 7;14(3):e0213459. doi: 10.1371/journal.pone.0213459 (PMC6405136; doi:10.1371/journal.pone.0213459)
Supplement: S2 Table — (DOCX) [file pone.0213459.s002.docx]

**S2 Table. List of the texture features extracted using the Pyradiomics and in-house Matlab-based softwares.**

| **Gray-Level Co-occurrence Matrix (GLCM)** | | |
| --- | --- | --- |
| **Feature name** | **Pyradiomics**  **(n = 23)** | **In-house software**  **(n = 26)** |
| **Autocorrelation** | ✔ | ✔ |
| **Joint Average** | ✔ |  |
| **Cluster Prominence** | ✔ | ✔ |
| **Cluster Shade** | ✔ | ✔ |
| **Cluster Tendency** | ✔ | ✔ |
| **Contrast** | ✔ | ✔ |
| **Correlation** | ✔ | ✔ |
| **Difference Average** | ✔ |  |
| **Difference Entropy** | ✔ | ✔ |
| **Difference Variance** | ✔ |  |
| **Dissimilarity** |  | ✔ |
| **Joint Energy** | ✔ | ✔ |
| **Joint Entropy** | ✔ | ✔ |
| **Homogeneity 1** |  | ✔ |
| **Homogeneity 2** |  | ✔ |
| **Measure of Information Correlation 1** | ✔ | ✔ |
| **Measure of Information Correlation 2** | ✔ | ✔ |
| **Inverse Difference Moment (IDM)** | ✔ |  |
| **Inverse Difference Moment Normalized (IDMN)** | ✔ | ✔ |
| **Inverse Difference** | ✔ |  |
| **Inverse Difference Normalized (IDN)** | ✔ | ✔ |
| **Inverse Variance** | ✔ | ✔ |
| **Maximum Probability** | ✔ | ✔ |
| **Sum Average** | ✔ | ✔ |
| **Sum Variance** |  | ✔ |
| **Sum Entropy** | ✔ | ✔ |
| **Sum Squares** | ✔ |  |
| **Variance1** |  | ✔ |
| **Variance2** |  | ✔ |
| **Correlation2** |  | ✔ |
| **Sum Average 2** |  | ✔ |
| **Agreement** |  | ✔ |
| **Gray-Level Size Zone Matrices (GLSZM)** | | |
| **Feature name** | **Pyradiomics**  **(n = 16)** | **In-house software**  **(n = 13)** |
| **Small Area Emphasis (SAE)** | ✔ | ✔ |
| **Large Area Emphasis (LAE)** | ✔ | ✔ |
| **Gray Level Non-Uniformity (GLN)** | ✔ | ✔ |
| **Gray Level Non-Uniformity Normalized (GLNN)** | ✔ |  |
| **Size-Zone Non-Uniformity (SZN)** | ✔ | ✔ |
| **Size-Zone Non-Uniformity Normalized (SZNN)** | ✔ |  |
| **Zone Percentage (ZP)** | ✔ | ✔ |
| **Gray Level Variance (GLV)** | ✔ | ✔ |
| **Zone Variance (ZV)** | ✔ | ✔ |
| **Zone Entropy (ZE)** | ✔ |  |
| **Low Gray Level Zone Emphasis (LGLZE)** | ✔ | ✔ |
| **High Gray Level Zone Emphasis (HGLZE)** | ✔ | ✔ |
| **Small Area Low Gray Level Emphasis (SALGLE)** | ✔ | ✔ |
| **Small Area High Gray Level Emphasis (SAHGLE)** | ✔ | ✔ |
| **Large Area Low Gray Level Emphasis (LALGLE)** | ✔ | ✔ |
| **Large Area High Gray Level Emphasis (LAHGLE)** | ✔ | ✔ |
| **Gray-Level Run Length Matrices (GLRLM)** | | |
| **Feature name** | **Pyradiomics**  **(n = 16)** | **In-house software**  **(n = 13)** |
| **Short Run Emphasis (SRE)** | ✔ | ✔ |
| **Long Run Emphasis (LRE)** | ✔ | ✔ |
| **Gray Level Non-Uniformity (GLN)** | ✔ | ✔ |
| **Gray Level Non-Uniformity Normalized (GLNN)** | ✔ |  |
| **Run Length Non-Uniformity (RLN)** | ✔ | ✔ |
| **Run Length Non-Uniformity Normalized (RLNN)** | ✔ |  |
| **Run Percentage (RP)** | ✔ | ✔ |
| **Gray Level Variance (GLV)** | ✔ | ✔ |
| **Run Variance (RV)** | ✔ | ✔ |
| **Run Entropy (RE)** | ✔ |  |
| **Low Gray Level Run Emphasis (LGLRE)** | ✔ | ✔ |
| **High Gray Level Run Emphasis (HGLRE)** | ✔ | ✔ |
| **Short Run Low Gray Level Emphasis (SRLGLE)** | ✔ | ✔ |
| **Short Run High Gray Level Emphasis (SRHGLE)** | ✔ | ✔ |
| **Long Run Low Gray Level Emphasis (LRLGLE)** | ✔ | ✔ |
| **Long Run High Gray Level Emphasis (LRHGLE)** | ✔ | ✔ |
| **Gray-Level Dependence Matrices (GLDM)** | | |
| **Feature name** | **Pyradiomics**  **(n = 14)** | **In-house software**  **(n = 0)** |
| **Small Dependence Emphasis (SDE)** | ✔ |  |
| **Large Dependence Emphasis (LDE)** | ✔ |  |
| **Gray Level Non-Uniformity (GLN)** | ✔ |  |
| **Gray Level Non-Uniformity Normalized (GLNN)** | ✔ |  |
| **Dependence Non-Uniformity (DN)** | ✔ |  |
| **Dependence Non-Uniformity Normalized (DNN)** | ✔ |  |
| **Gray Level Variance (GLV)** | ✔ |  |
| **Dependence Variance (DV)** | ✔ |  |
| **Dependence Entropy (DE)** | ✔ |  |
| **Dependence Percentage** | ✔ |  |
| **Low Gray Level Emphasis (LGLE)** | ✔ |  |
| **High Gray Level Emphasis (HGLE)** | ✔ |  |
| **Small Dependence Low Gray Level Emphasis (SDLGLE)** | ✔ |  |
| **Small Dependence High Gray Level Emphasis (SDHGLE)** | ✔ |  |
| **Neighboring Gray Tone Difference Matrices (NGTDM)** | | |
| **Feature name** | **Pyradiomics**  **(n = 0)** | **In-house software**  **(n = 5)** |
| **Coarseness** |  | ✔ |
| **Contrast** |  | ✔ |
| **Busyness** |  | ✔ |
| **Complexity** |  | ✔ |
| **Strength** |  | ✔ |
