# Supplementary material for: Gray-level discretization impacts reproducible MRI radiomics texture features
Source: PLoS One. 2019 Mar 7;14(3):e0213459. doi: 10.1371/journal.pone.0213459 (PMC6405136; doi:10.1371/journal.pone.0213459)
Supplement: S3 Table — (DOCX) [file pone.0213459.s003.docx]

**S3 Table. Feature extraction details according to the Imaging Biomarker Standardization Initiative (IBSI) guidelines.**

| **General** | | |
| --- | --- | --- |
|  | **Dataset 1** | **Dataset 2** |
| **Imaging** | MRI | |
| **Acquisition** | See Supplementary Table 1 | |
| **Approach** | 2D | |
| **Process workflow** | See Figure 1 | |
| **Software** | Pyradiomics (experiment 1)  In-house software (experiment 2) | Pyradiomics |
| **Data availability** | Images: not available  Software: Pyradiomics ([https://pyradiomics.readthedocs.io/en/latest/#](https://pyradiomics.readthedocs.io/en/latest/)) / In-house software: no available | |
| **Data Conversion** | | |
| **Procedure** | Conversion from DICOM format to Nifti format | |
| **Image Post-Acquisition Processing** | | |
| **Procedure** | None | |
| **Segmentation** | | |
| **ROI procedure** | Lacrymal glands | Breast tumors |
|  | Two readers, total of three readings (1 by reader 1, 2 by reader 2) | |
|  | Manual delineation  + computer simulations of delineation’s variability (experiment 4) | |
| **Interpolation** | | |
| **Voxel dimensions (mm)** | ADC map: 0.8x0.8x3  T1-WI: 0.375x0.375x2.5  DIXON-T2-WI : 0.33 x 0.33 x 2  PC DIXON-T1-WI : 0.44 x 0.44 x 3.8 | DISCO : 0.68 x 0.68 x 1 |
| **Image interpolation method** | None (2D textures on isotropic images) | |
| **Re-segmentation** | | |
| **ROI mask criteria** | Not applicable |  |
| **Discretization** | | |
| **Discretization methods** | Fixed Bin Size (FBS) and Fixed Bin Number (FBN) | |
| **Discretization parameters** | FBS: bin widths = 1, 5, 10, 20, 25, 50  FBN: bin numbers = 8, 16, 32, 64, 128, 256, 512, 1024 | |
| **Feature calculation** | | |
| **Feature set** | See Supplementary Table 2 | |
| **Feature parameters** | **Distance setting:** 1 pixel  **Calculation methods**  *Pyradiomics:* mean of texture values obtained from each normalized matrices in each direction  *In-house software:* one texture value computed on a unique non normalized merged matrix | |
