# Supplementary material for: Gray-level discretization impacts reproducible MRI radiomics texture features
Source: PLoS One. 2019 Mar 7;14(3):e0213459. doi: 10.1371/journal.pone.0213459 (PMC6405136; doi:10.1371/journal.pone.0213459)
Supplement: S4 Table — Reproducible features using the Pyradiomics software and manual delineations on DATASET 1 according to the gray-level discretization. The intensity normalization was performed as follows: images were centered by their mean and scaled by their standard deviation; intensity values below or above three standard deviations from the mean were excluded; intensity values were shifted to get only positive values; intensities were then scaled to the initial common range (all images of each sequence had a similar range). The normalization was not applied to the parametric ADC map. (DOCX) [file pone.0213459.s004.docx]

**S4 Table. Experiment 1 results after intensity normalization. Reproducible features using the Pyradiomics software and manual delineations on DATASET 1 according to the gray-level discretization.** The intensity normalization was performed as follows: images were centered by their mean and scaled by their standard deviation; intensity values below or above three standard deviations from the mean were excluded; intensity values were shifted to get only positive values; intensities were then scaled to the initial common range (all images of each sequence had a similar range). The normalization was not applied to the parametric ADC map.

| **Sequence** | **Difference in reproducible features distribution (p-value)** | | **Highest number of reproducible features** | | |
| --- | --- | --- | --- | --- | --- |
|  | **Among FBS** | **Among FBN** | **Number** | | **P-Value** |
|  |  |  | **FBS** | **FBN** |  |
| **wDIXON-T2-WI** | 0.64 | **< 0.001*** | **52** | 34 | **< 0.01*** |
| **ipDIXON-T2-WI** | 0.23 | **< 0.01*** | **48** | 32 | **< 0.05*** |
| **pc wDIXON-T1-WI** | 0.83 | **< 0.05*** | **37** | 34 | 0.73 |
| **pc ipDIXON-T1-WI** | 1 | **< 0.01*** | **37** | 35 | 0.86 |
| **T1-WI** | 0.48 | **< 0.01*** | **21** | 21 | 1 |
| **ADC map** | 1 | 0.41 | **31** | 9 | **< 0.001*** |

FBS= Fixed Bin Size (absolute discretization), FBN= Fixed Bin Number (relative discretization). ***Sequence abbreviations:*** Water DIXON-T2-WI (wDIXON-T2-WI); In-Phase DIXON-T2-WI (ipDIXON-T2-WI); Post-Contrast Water DIXON-T1-WI (pc wDIXON-T1-WI); Post-Contrast In-Phase DIXON-T1-WI (pc ipDIXON-T1-WI); Apparent Diffusion Coefficient map (ADC map). * = statistically significantly different among discretizations.
