# Supplementary material for: Gray-level discretization impacts reproducible MRI radiomics texture features
Source: PLoS One. 2019 Mar 7;14(3):e0213459. doi: 10.1371/journal.pone.0213459 (PMC6405136; doi:10.1371/journal.pone.0213459)
Supplement: S5 Table — Pyradiomics computes one matrix per distance and/or angle (according to the texture feature), normalizes the matrix, calculates one feature value per normalized matrix and averages the results to obtain the final texture value. Our in-house software computes a weighted sum of all non-normalized matrices obtained with the different distances/angles (diagonals are weighted by √2), then directly calculates the final feature value based on this unique matrix. These two methods are described in the IBSI guidelines. There is no current recommendation to favor one method over another. (DOCX) [file pone.0213459.s005.docx]

**S5 Table. Pyradiomics versus in-house software texture features calculation methods.** Pyradiomics computes one matrix per distance and/or angle (according to the texture feature), normalizes the matrix, calculates one feature value per normalized matrix and averages the results to obtain the final texture value. Our in-house software computes a weighted sum of all non-normalized matrices obtained with the different distances/angles (diagonals are weighted by $\surd2$), then directly calculates the final feature value based on this unique matrix. These two methods are described in the IBSI guidelines. There is no current recommendation to favor one method over another.

| **Texture features calculation methods** | |
| --- | --- |
| **Pyradiomics** | **In-house software** |
| One matrix per angle/distance  ↓  Normalization of each matrix  ↓  One feature value per matrix  ↓  Average of feature values  =  Final Value | One matrix per angle/distance  ↓  Weighted sum of all matrices (not normalized)  ↓  One feature value  =  Final Value |
